# Ancient Schwannoma Along the Patellar Tendon: Unveiling a Rare Clinical Phenomenon With Literature Review

**DOI:** 10.7759/cureus.63295

**Published:** 2024-06-27

**Authors:** Siddhant Pundalik Pol, Sunil K Bhosale, Jigar Desai, Dipen Ariwala, Chintan Vaidya, Shahrukh Azad, Falgun Dhawale, Navnath Jawale

**Affiliations:** 1 Department of Orthopedics, Seth Gordhandas Sunderdas Medical College and King Edward Memorial Hospital, Mumbai, IND

**Keywords:** orthopedics surgery, knee joint schwannoma, extra-articular tumor, infra-patellar swelling, schwannoma

## Abstract

Ancient schwannoma, a rare subtype of schwannoma, a benign tumor originating from nerve sheaths, can arise from various nerves, except for the optic, olfactory, spinal, and autonomic nervous systems. Schwannomas are typically characterized by the presence of neoplastic Schwann cells and tend to develop eccentrically. Malignant transformations of schwannomas are exceptionally uncommon.

In this case report, a 42-year-old male presented with a painful lump on the front of his left knee. The lump was described as an extra-articular swelling below the kneecap, situated over the patellar tendon. Initially, ultrasonography (USG) indicated the presence of a slow-flow vascular malformation in the infrapatellar region of the left knee. However, subsequent magnetic resonance imaging (MRI) revealed a well-defined mass in the subcutaneous plane below the knee, with minimal septations, leading to an initial suspicion of a large sebaceous cyst.

Further investigation through histopathological analysis confirmed the diagnosis of an extra-articular schwannoma. This finding highlights the importance of thorough examination and diagnostic techniques in differentiating between various types of soft tissue masses. Schwannomas, although uncommon in certain locations, should be considered in the differential diagnosis of painful lumps, even in atypical anatomical sites such as the knee.

## Introduction

Schwannomas, which are noncancerous tumors that arise from the peripheral nerves, are a prevalent type of neoplasms that impact these structures [1]. They are usually enclosed, occurring alone, and frequently exhibit an unusual placement along the nerve sheath. These tumors are mainly made up of Schwann cells and have a unique histological pattern. This pattern is characterized by being enclosed and having two distinct areas called Antoni A and B. Antoni A regions exhibit a high concentration of spindle cells, whereas Antoni B regions have lower cell density and may include fluid-filled spaces [2]. The occurrence of malignant transformation in schwannomas is extremely rare, happening in less than 1% of cases. Although these tumors are typically harmless, they can present symptoms such as pain, swelling, or the detection of a palpable lump. Nevertheless, they commonly display gradual, symptomless expansion and are frequently discovered unintentionally during regular check-ups or imaging procedures. Schwannomas display distinctive histological features that facilitate their diagnosis. The differentiation between Antoni A and B regions offers a valuable understanding of the cellular composition and architectural arrangement of the tumor, aiding in precise identification and categorization [3]. Curiously, although they are common, there have been only a few documented instances of extra-articular schwannomas impacting the knee [4]. The limited availability emphasizes the significance of acknowledging and comprehending the various manifestations of these tumors, especially in atypical anatomical sites. The infrequency of such events underscores the necessity for heightened consciousness among orthopedic experts to guarantee prompt identification and suitable treatment. The purpose of this case report is to bring attention to the possible contribution of extra-articular schwannomas to knee conditions. Through emphasizing this extraordinary instance, our aim is to enhance the overall knowledge and comprehension of schwannoma manifestation and treatment, specifically in unconventional anatomical locations such as the knee.

## Case presentation

**Figure 1:**
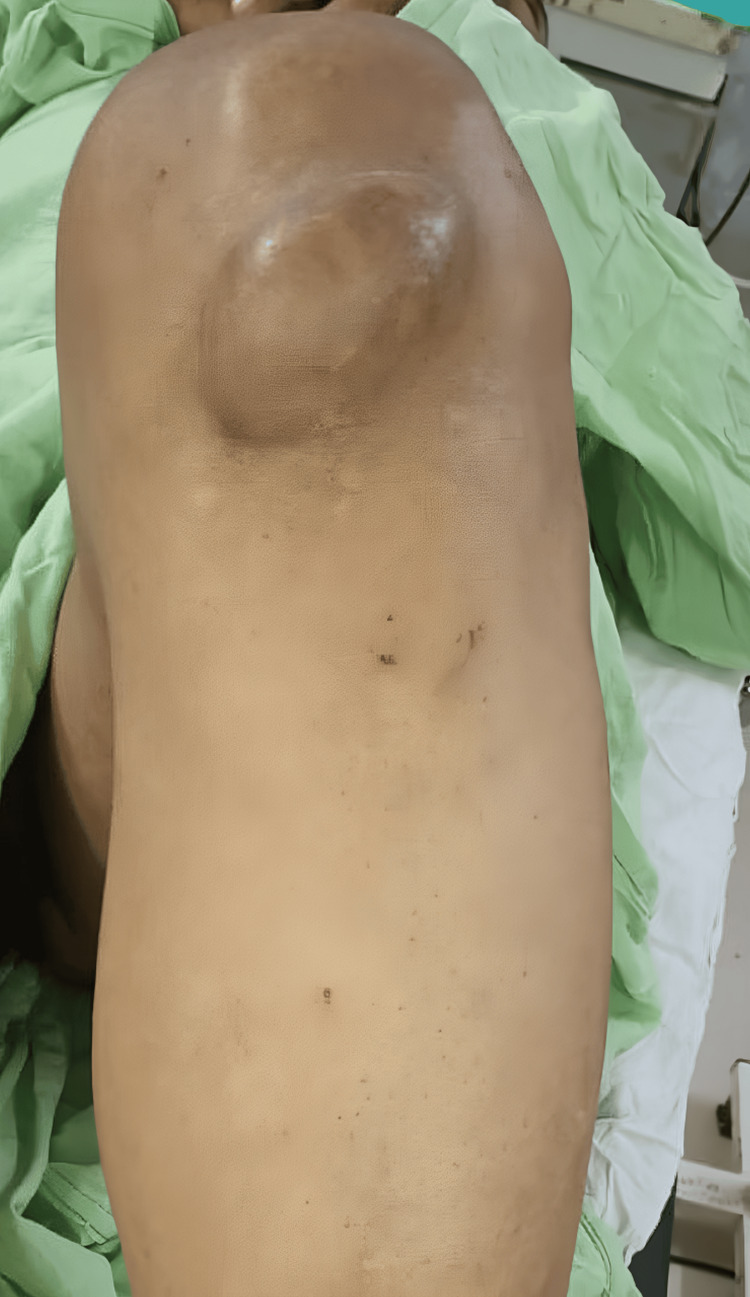
Preoperative image showing the lesion at the knee

A 42-year-old man, who was previously in good health with no known medical conditions, came to the doctor with a long-lasting issue of swelling in his left knee that had been going on for 12 years. The appearance of symptoms coincided with a traumatic incident that took place 12 years ago. During the examination, a clearly detectable solid lump was found in the lower part of the kneecap on the front side. The swelling exhibited characteristics such as a soft texture, the ability to fluctuate in size, no adherence to the tissues beneath it, and no changes in the skin. Pain caused by palpation was noted, but no restrictions were observed in the knee joint's range of motion (Figure 1).

**Figure 2:**
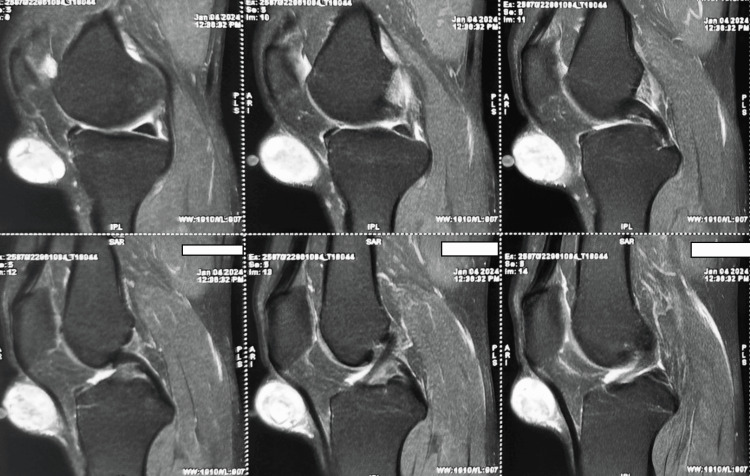
Sagittal T2- and T1-weighted MRI showing an infrapatellar sebaceous cyst MRI, magnetic resonance imaging

The initial ultrasonography (USG) indicated the existence of a slow-flow vascular malformation located below the kneecap on the left knee. The dimensions of the swelling were measured to be 4x3x2 cm in diameter. The follow-up magnetic resonance imaging (MRI) showed a lesion in the infrapatellar subcutaneous plane that appeared dark on T1-weighted images and bright on T2-weighted images. The lesion was also observed to have very thin divisions. Based on these imaging findings, there was initial consideration of a sizable sebaceous cyst (Figure 2).

**Figure 3:**
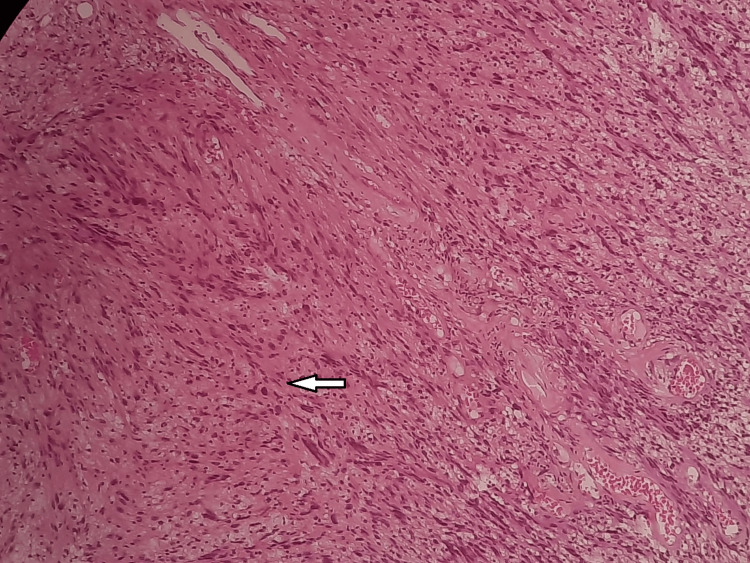
Fibrillar aspects and palisades of elongated spindle cells (hematoxylin and eosin staining) The arrow shows fibrillar aspect of the lesion.

A surgical excision was planned and then the excision was sent for histopathological reporting, which shows fibrillar aspects and palisades of elongated spindle cells (hematoxylin and eosin staining) (Figure 3).

The surgical approach for removing the infrapatellar lesion in the left knee involved a longitudinal incision to provide optimal access and visualization of the area. The procedure aimed for a total resection, ensuring that the entire lesion was excised. Histopathological analysis of the excised specimen, which revealed fibrillar aspects and palisades of elongated spindle cells stained with hematoxylin and eosin, confirmed the diagnosis of a schwannoma. Importantly, the histopathology report indicated that free edges were achieved, meaning no residual tumor tissue was left at the margins, thus minimizing the risk of recurrence and confirming the success of the complete removal.

## Discussion

According to a study conducted by Dean et al. in 2011, pigmented villonodular synovitis was found to be the most common diagnosis among 19 patients with benign tumors inside the joints [5]. In 2013, Albergo et al. conducted a study on 25 patients with pigmented villonodular synovitis and hemangioma being the most frequently diagnosed conditions. It is worth mentioning that no cases of schwannoma were found among these patients [6]. Although schwannoma is uncommon, it should be considered a possible diagnosis [7]. This case study provides insight into the diagnostic procedure for identifying a non-malignant tumor that originates from the peripheral nervous system and is located outside of the joint [8]. 

Schwannomas commonly occur in individuals in their 40s and 50s, with a slightly higher occurrence in females at a ratio of 1.6:1 [9]. Because of their sluggish rate of growth, they frequently go unnoticed and are inadvertently stumbled upon. In addition, they are infrequent in the lower limbs, with only one documented instance of a schwannoma within the knee joint reported in the literature [5]. The median nerve was identified as the most common site for schwannomas. These peripheral schwannomas can originate from either motor or sensory nerves [10]. An extensive assessment of the patient's symptoms, medical history, and physical examination findings yielded the presence of a tumor. Microscopic examination was necessary to definitively confirm the diagnosis of schwannoma. Before the surgery, radiographs and MRIs were instrumental in assessing the tumor's size and scope. Based on the patient's symptoms, it was advised and promptly carried out to perform a significant surgical removal. MRI reveals the location, size, texture, and relationships with surrounding neuromuscular structures [11]. MRI has surpassed radiography as the definitive method for accurate diagnosis, while radiography still plays a valuable role in initial screening to rule out malignant tumors [7]. When there is only one tumor, intervention is usually prompted by the symptoms that appear. Probably marginal resection along with pathological examination for correct diagnosis [12].

## Conclusions

This case represents the first documentation of an infrapatellar extra-articular schwannoma of the knee, which adds a valuable contribution to the limited literature on this rare tumor. In summary, it underscores the importance of considering schwannoma as a potential differential diagnosis in cases of extra-articular tumors affecting peripheral nerves, especially in the lower limbs. Despite its rarity, schwannoma can present with symptoms such as pain and swelling, underscoring the need for thorough clinical evaluation. The successful diagnosis and management of this case emphasize the importance of meticulous history-taking, comprehensive physical examination, and appropriate imaging modalities, such as MRI, in confirming the diagnosis and guiding treatment decisions. Surgical excision remains the cornerstone of treatment for solitary schwannomas, ensuring complete removal and reducing the risk of recurrence.

By presenting this case, our aim is to enrich the existing literature on schwannomas and raise awareness among clinicians, particularly those specializing in orthopedics and oncology, about this uncommon yet clinically significant entity. Further studies and case reports are warranted to deepen our understanding of schwannomas and refine their management strategies.
